# Ultrasonographic evaluation demonstrating safer needle–organ distances in a novel quadratus lumborum block technique in cats

**DOI:** 10.3389/fvets.2026.1761450

**Published:** 2026-01-26

**Authors:** Fumihiko Takusagawa, Yoshimi Takusagawa, Taro Kimura

**Affiliations:** 1 Seibozaka Animal Hospital, Tokyo, Japan; 2 Vet Surg Tokyo, Tokyo, Japan

**Keywords:** feline, needle trajectory, quadratus lumborum block, regional anesthesia, ultrasound-guided nerve block

## Abstract

**Introduction:**

We investigated the relationship between needle placement and adjacent organs using ultrasonography during a novel quadratus lumborum block (QLB) performed in sternal recumbency and the conventional QLB performed in lateral recumbency among cats undergoing elective ovariohysterectomy.

**Methods:**

Fourteen client-owned female cats were randomly allocated to the conventional QLB group (*n* = 7) or the novel QLB group (*n* = 7). For the conventional QLB group, the ultrasound probe was positioned at the second lumbar vertebra, whereas for the novel QLB group, it was placed at the intertransverse space between the second and third lumbar vertebrae. Probe angle was standardized at 90° using a 180° protractor, and tilted views were obtained at 70°, 80°, 100°, and 110°. The 90° image served as the needle insertion reference. OsiriX was used to identify organs within 5 mm of the needle shaft and to measure distances from the needle shaft, entry point, and needle tip to the nearest organs on the 90° image. Distances from a hypothetical entry point projected onto the tilted images were also evaluated.

**Results:**

Compared with the conventional QLB group, the novel QLB group had fewer organs within 5 mm of the needle shaft on the right side. The novel QLB group also showed greater distances from the needle shaft and entry point to the nearest organs, as well as a shorter distance from the needle tip to the nearest organ. In tilted images, no between-group differences were identified except at 100° on the left side, where the novel QLB group had a longer distance. Blood vessels were the structures most frequently identified near the needle pathway.

**Discussion:**

The novel QLB positioned the needle trajectory farther from vital organs, particularly on the right side, supporting its potential safety advantage. Real-time ultrasonographic visualization remains necessary because organs were still identified parallel to the trajectory or distal to the needle tip.

## 1 Introduction

Regional anesthesia has gained increasing prominence in contemporary veterinary anesthesia as an essential component of perioperative pain management (1, 2). The quadratus lumborum block (QLB) is a regional anesthetic technique that deposits local anesthetic around the quadratus lumborum muscle, allowing spread to the spinal nerves and sympathetic trunk and providing somatic and visceral analgesia, respectively (3). This technique has demonstrated effectiveness in attenuating nociception associated with celiotomy in cats (4–7) and dogs (8–10). The conventional QLB (CQLB) is performed in lateral recumbency, during which the needle is advanced in a ventrodorsal direction that passes near critical organs such as the kidney, spleen, and aorta, increasing the potential risk of inadvertent organ injury (11, 12). We recently described a novel QLB (NQLB) technique performed in sternal recumbency, which enables the needle trajectory to avoid close proximity to vital organs and reduces isoflurane requirements during feline ovariohysterectomy (13). Despite these advantages, the safety profile of this novel approach has not yet been fully established. Therefore, the aim of the present study was to objectively compare the spatial relationship between the needle path and adjacent or distal vital organs, as an indicator of safe needle placement, between the CQLB and NQLB techniques using ultrasonographic assessment. The primary outcome was the proportion of vital organs located within 5 mm of the needle shaft. The secondary outcomes were as follows: (1) the distances from the needle shaft, needle entry point on the ultrasonographic image, and needle tip to each vital organ and (2) the distance from the hypothetical needle entry point, which was extrapolated from the standard ultrasonographic image, to the nearest vital organ on ultrasonographic images obtained by tilting the probe to simulate an incorrect needle trajectory. We hypothesized that the proportion of vital organs located close to the needle shaft would be lower in the NQLB than in the CQLB. Additionally, we hypothesized that the distances between vital organs and the needle (actual needle position) or the hypothetical needle entry point (derived from tilted images) would be greater in the NQLB than in the CQLB.

## 2 Materials and methods

### 2.1 Study design and animals

This prospective clinical trial with sequential enrollment was approved by the Institutional Animal Care and Use Committee of Kimura Animal Hospital (approval No. KAH2025-01). Informed consent was obtained from the owners of all enrolled cats.

The sample size was calculated based on data obtained from a pilot study. In the pilot data, the proportion of cases in which a vital organ was located within 5 mm of the needle shaft on the right side was 0% in the NQLB group and 80% in the CQLB group. Assuming a two-sided α level of 0.05 and a statistical power of 0.90, the required sample size was calculated as six cats per group. To account for a potential dropout rate of approximately 10%, seven cats per group were enrolled in the present study. Accordingly, we included 14 client-owned cats classified as American Society of Anesthesiologists Physical Status I and with a body condition score of three–seven who were scheduled for elective ovariohysterectomy. We excluded cats with lumbar anatomical abnormalities, local infection at the injection site, or a history of adverse reactions to local anesthetics. A random sequence of seven 0s (CQLB group) and seven 1s (NQLB group) was generated using Random.org (Randomness and Integrity Services Ltd., Dublin, Ireland), which was subsequently consecutively assigned to cats based on the order of hospital admission. Next, the cats were allocated to the corresponding groups.

### 2.2 Anesthesia and monitoring

All cats received maropitant (Cerenia Injectable Solution; Zoetis Japan Co., Ltd., Tokyo, Japan; 1.0 mg/kg subcutaneously) upon admission. They underwent premedication with intramuscular alfaxalone (Alfaxan Multidose Injection; Jurox Pty Ltd., Rutherford, NSW, Australia; 1.0 mg/kg), midazolam (Dormicum Injection; Astellas Pharma Inc., Tokyo, Japan; 0.2 mg/kg), and dexmedetomidine (Dexdomitor Injection; Orion Corporation, Espoo, Finland; 4 μg/kg), which were combined in the same syringe. After 10 min, an intravenous catheter (Surflo Flash 24-gauge, 3/4 inch; Terumo Corporation, Tokyo, Japan) was placed in the medial saphenous vein. Next, intravenous alfaxalone (median dose, 1.0 mg/kg; range, 0.5–2.0 mg/kg) was administered to induce anesthesia, followed by tracheal intubation using an appropriately sized cuffed endotracheal tube (Spiral Endotracheal Tube with Stylet and Cuff, 3.5–4.0 mm internal diameter; Fuji Systems Corporation, Tokyo, Japan) upon confirmation of loss of the swallowing reflex and jaw tone. Subsequently, the endotracheal tube was connected to a rebreathing circle system via an anesthetic machine (Acoma Veterinary Anesthesia Machine FO-20A; ACOMA Medical Industry Co., Ltd., Tokyo, Japan). Anesthesia was maintained using isoflurane (Isoflurane for Animal Use; Riken Animal Health Co., Ltd., Tokyo, Japan) in 100% oxygen. The vaporizer setting was adjusted to maintain an end-tidal isoflurane concentration of approximately 1.4%. After intubation, cefazolin [Cefazolin Injection “Fujita”; Fujita Pharmaceutical Co., Ltd., Tokyo, Japan (25 mg/kg intravenous)] and meloxicam [Inflacam 0.5% Injection; Virbac Japan Co., Ltd., Osaka, Japan (0.3 mg/kg subcutaneous)] were administered, followed by infusion of lactated Ringer's solution (Solulact Infusion 250 ml; Terumo Corporation) at 5 ml/kg/h. Monitoring included continuous electrocardiography, pulse oximetry, capnography, and esophageal temperature measurement, with recording of noninvasive oscillometric blood pressure at 2-min intervals using a multiparameter monitor (ePM12M Vet; Mindray, Shenzhen, China).

### 2.3 Quadratus lumborum block and data acquisition

All QLB procedures were performed by the same veterinary anesthetist (FT) using a 22-gauge, 70-mm echogenic needle (Sonolect Needle; Hakko Co., Ltd., Chikuma, Japan) and a dedicated syringe (TOP 2.5 ml ISO 80369-6-compliant syringe; TOP Corporation, Tokyo, Japan) filled with 0.125% bupivacaine (Bupivacaine Hydrochloride Injection 0.25%; Maruishi Pharmaceutical Co., Ltd., Osaka, Japan). Bupivacaine was administered at a total volume of 0.8 ml/kg (0.4 ml/kg per hemiabdomen), followed by an additional 0.4 ml used to prime the extension tubing.

**Figure 1:**
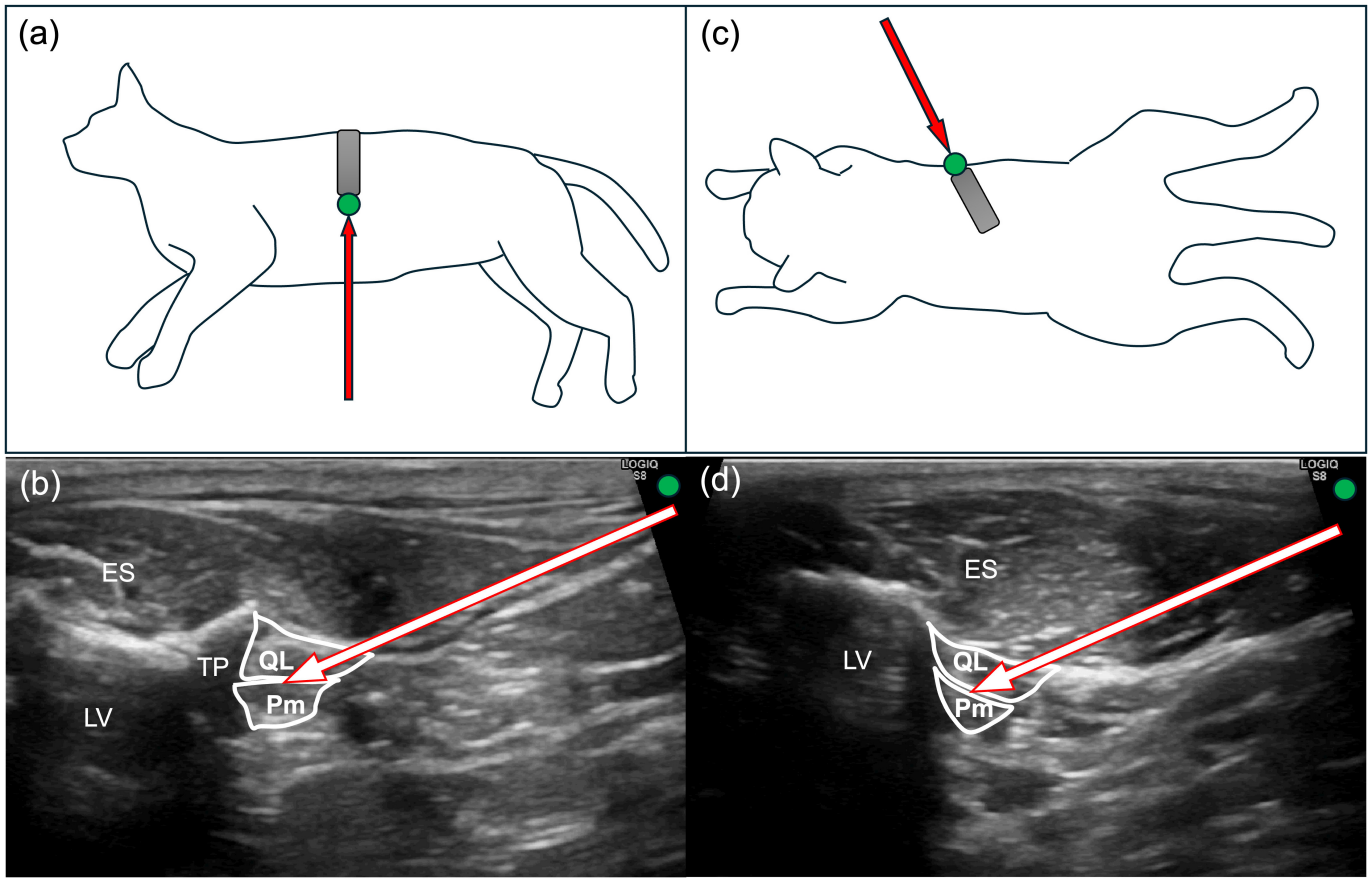
Diagram of probe positioning, needle insertion direction, and corresponding ultrasonographic images for the conventional and novel quadratus lumborum block (QLB) in cats. **(a)** Probe positioning (gray rectangle) with the reference mark (green circle) in the conventional QLB. A red arrow indicates the needle insertion direction. **(b)** Ultrasonographic image corresponding to **(a)** depicting the needle trajectory. **(c)** Probe positioning (gray rectangle) with the reference mark (green circle) in the novel QLB. A red arrow indicates the needle insertion direction. **(d)** Ultrasonographic image corresponding to **(c)** depicting the needle trajectory. ES, erector spinae muscle; LV, lumbar vertebra; Pm, psoas minor muscle; QL, quadratus lumborum muscle; TP, transverse process.

**Figure 2:**
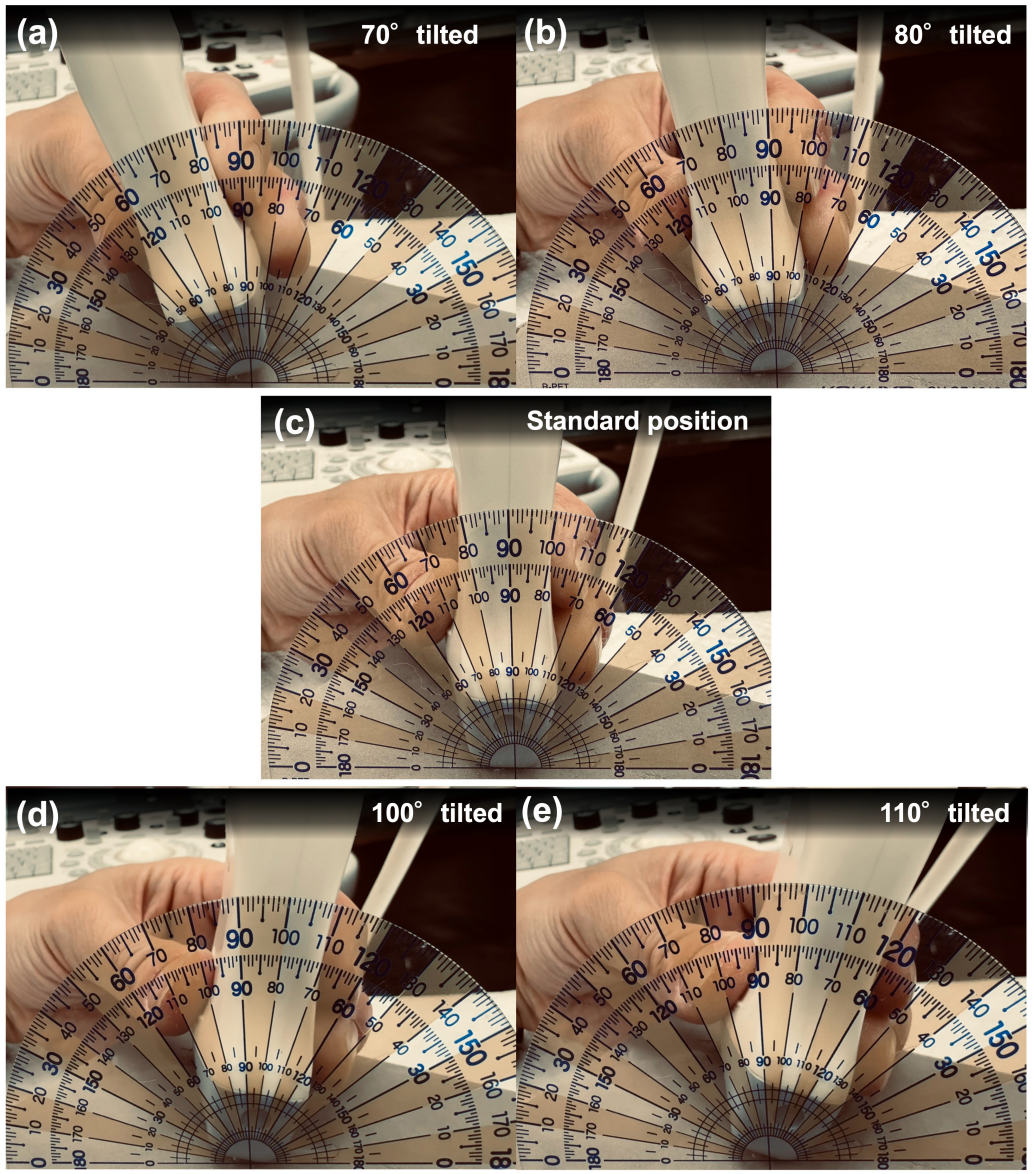
Illustrative representation of the 180° protractor positioned relative to the ultrasound probe. **(a)** The probe tilted at 70°, corresponding to 20° cranial to the standard position. **(b)** The probe tilted at 80°, corresponding to 10° cranial to the standard position. **(c)** The probe positioned at the standard (90°) angle for needle insertion. **(d)** The probe tilted at 100°, corresponding to 10° caudal to the standard position. **(e)** The probe tilted at 110°, corresponding to 20° caudal to the standard position.

In the CQLB group, the cats were positioned in left lateral recumbency. The hair over the right dorsolumbar region, extending from the caudal border of the last rib to the level of the fourth lumbar vertebra and from the dorsal midline to the ventrolateral abdominal wall, was clipped, followed by aseptic preparation. A linear ultrasound probe (11 L-D Linear Array Transducer; GE Healthcare, Chicago, IL, United States) with a frequency of 12 MHz, connected to an ultrasound machine (LOGIQ S8; GE Healthcare), was placed perpendicular to the longitudinal axis of the body to obtain a transverse ultrasonographic view, with the reference mark directed ventrally (Figure 1a). The transverse process of the second lumbar vertebra and the quadratus lumborum and psoas minor muscles were identified on the ultrasonographic image with the reference mark positioned on the right side of the screen (Figure 1b). The ultrasonographic image in which the target needle tip location, namely the interfascial plane between the quadratus lumborum and psoas minor muscles ventral to the second lumbar transverse process, was clearly visualized was defined as the optimal image for needle insertion. After acquisition of this optimal image, a 180° protractor (Manabist Protractor, Recycled PET, 90 × 50 mm, semi-circular, two-piece set; Kokuyo Co., Ltd., Osaka, Japan) was positioned along the dorsal edge of the probe at 90° (Figure 2c). Subsequently, the probe was tilted to 70°, 80°, 100°, and 110° (Figures 2a, b, d, e), with each tilted image stored. After each image acquisition, the probe was repositioned to the 90° orientation for needle insertion, and the protractor was reset to ensure consistent alignment. An in-plane ventrodorsal needle insertion was performed under ultrasonographic guidance on the 90° image, with the needle tip positioned in the interfascial plane between the quadratus lumborum and psoas minor muscles at the time of injection, and the complete needle trajectory was recorded. After confirming a negative blood aspiration, the local anesthetic agent was injected. Subsequently, the same procedure, including image acquisition and negative aspiration followed by local anesthetic injection, was repeated on the contralateral side in left lateral recumbency.

In the NQLB group, the cats were positioned in sternal recumbency. The hair over the bilateral dorsolumbar regions, extending from the caudal border of the last rib to the level of the fourth lumbar vertebra and from the dorsal midline to the ventrolateral abdominal wall, was clipped, followed by aseptic preparation. The ultrasound probe was positioned between the second and third lumbar transverse processes, aligned with the transverse process orientation (Figure 1c) and the lumbar vertebral body. The quadratus lumborum and psoas minor muscles were identified on the ultrasonographic image with the reference mark positioned on the right side of the screen (Figure 1d). The ultrasonographic image in which the target needle tip location, namely the interfascial plane between the quadratus lumborum and psoas minor muscles in direct contact with the lumbar vertebral body and without interposition of the transverse process along the anticipated needle trajectory, was clearly visualized on the 90° image was defined as the optimal image for needle insertion. Image acquisition and storage were performed as in the CQLB group, except that the protractor was positioned along the caudomedial edge of the probe, and needle insertion was performed in a craniolateral-to-caudomedial direction. An in-plane needle insertion was performed under ultrasonographic guidance on the 90° image, with the needle tip positioned in the interfascial plane between the quadratus lumborum and psoas minor muscles at the time of injection, and the complete needle trajectory was recorded. In the NQLB group, all procedures and image acquisitions were sequentially performed from right to left. For both groups, all ultrasonographic images were acquired and stored by the same veterinarian (YT).

After image acquisition and ≥10 min after completion of the QLB procedures, ovariohysterectomy was performed using a standard surgical technique under appropriate anesthesia and perioperative analgesia.

### 2.4 Image analysis and measurements

**Figure 3:**
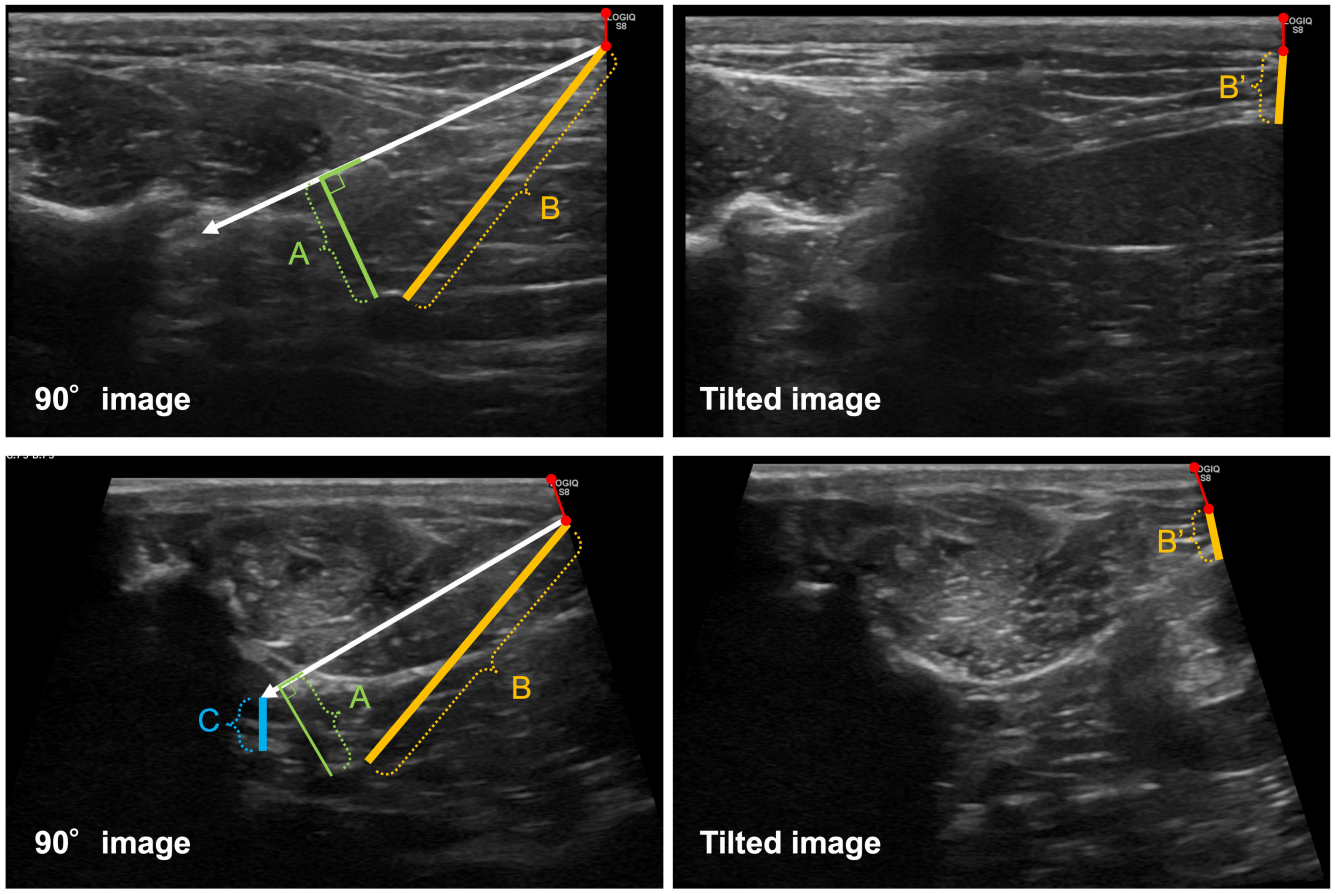
Diagrammatic representation of distance measurements between the needle and adjacent organs. A white arrow indicates the needle shaft. “A” indicates the distance between the needle shaft and the nearest organ on the 90° image, measured as the shortest perpendicular distance from the shaft. “B” indicates the distance between the needle entry point and the nearest vital organ on the 90° image. “C” indicates the distance between the needle tip and the nearest vital organ located distal to the tip on the 90° image. B′ indicates the distance between the hypothetical needle entry point (extrapolated from the actual entry point on the 90° image; red line with circles at both ends) and the nearest vital organ on the tilted images. The upper two panels show representative images obtained using the conventional quadratus lumborum block (CQLB), whereas the lower two panels show representative images obtained using the novel quadratus lumborum block (NQLB).

All ultrasonographic images were exported as DICOM files and analyzed using a DICOM viewer (OsiriX MD; Pixmeo SARL, Geneva, Switzerland). The liver, spleen, kidney, stomach, intestine, and major blood vessels (aorta and caudal vena cava, including their primary branches) were identified as the vital organs in the images as these structures are anatomically located along or in close proximity to the potential needle trajectory during quadratus lumborum block and inadvertent needle penetration of these organs may result in clinically relevant complications. In case a vital organ was identified, the distances described below were measured for each image (Figure 3).

For the image displaying the needle trajectory (90° image), we measured the distances from the needle shaft to the nearest vital organ, from the needle tip to the nearest vital organ distally, and from the actual needle entry point to the nearest vital organ.

For the tilted images (70°, 80°, 100°, and 110°), a hypothetical needle entry point was determined along the right edge of the image based on the relative position between the upper right corner of the ultrasonographic image and the actual needle entry site on the 90° image, followed by measurement of the distance from this point to the nearest vital organ.

All measurements were performed using the Region of Interest tool in OsiriX.

### 2.5 Outcomes and statistical analysis

We performed between-group comparisons of the proportion of cases in which a vital organ was located within 5 mm of the needle shaft in the 90° image. Additionally, we performed between-group comparisons of the distances between the needle shaft and nearest vital organ, between the needle tip and the nearest vital organ located distally, between the needle entry point and the nearest vital organ in the 90° image, and between the hypothetical needle entry point and the nearest vital organ in each tilted image. If no vital organ was visible in the image or distal to the needle tip, the corresponding image data were excluded from the distance comparison analysis. Furthermore, the identified nearest organ in each image was recorded and descriptively summarized to evaluate the frequency distribution of organs located proximally to the needle trajectory.

The Shapiro–Wilk test was used to assess the normality of continuous data. Normally and non-normally distributed data are reported as mean ± standard deviation and median (range), respectively. Between-group comparisons of continuous variables were performed using the unpaired *t-*test or Mann–Whitney *U*-test, depending on the normality of data distribution. Between-group comparisons of categorical data were performed using Fisher's exact test. Statistical analyses were performed using EZR (Saitama Medical Center, Jichi Medical University, Japan). Statistical significance was set at *p* < 0.05.

## 3 Results

Table 1 summarizes the demographic characteristics, including age, body weight, body condition score, and breed distribution. No significant between-group differences were observed in the demographic characteristics.

**Table 1:** Demographic characteristics of each group.

**Variable**	**CQLB (*n* = 7)**	**NQLB (*n* = 7)**
Age (months)	10.0 (6–123)	6.0 (3–138)
Body weight (kg)	2.80 ± 0.51	2.73 ± 0.26
BCS (1–9)	5 (4–6)	5 (5–6)
Breed	American Shorthair (1) Devon Rex (2) Mix (1) Munchkin (1) Scottish Fold (1) Siberian (1)	Devon Rex (1) Mix (4) Munchkin (1) Scottish Fold (1)

Data are presented as mean ± standard deviation or median (range), as appropriate. Breed data are presented with the number of cats (n) in parentheses. BCS, body condition score; CQLB, conventional quadratus lumborum block; NQLB, novel quadratus lumborum block.

The number of cases in which a vital organ was located within 5 mm of the needle shaft on the 90° image on the right side was 0/7 (0.0%) and 5/7 (71.4%) in the NQLB and CQLB groups, respectively, while the corresponding values for the left side were 1/7 (14.3%) and 3/7 (42.9%). This indicated a significant between-group difference on the right side (*p* = 0.021) but not for the left side (*p* = 0.559).

Table 2 summarizes all results regarding the distances from the needle shaft, entry point, and tip to the nearest vital organs. All 90° images in the CQLB group and three images in the NQLB group on the right side contained at least one vital organ near the needle shaft. The mean distance from the needle shaft to the nearest vital organ was significantly shorter in the CQLB group than in the NQLB group on the right side (*p* = 0.006). On the left side, we did not perform a comparative analysis because vital organs were identified in five and one image in the CQLB and NQLB groups, respectively.

**Table 2:** Number of images in which vital organs were detected and the distance from the needle shaft, needle entry point, and needle tip on the 90° ultrasonographic image.

**Detection site of the nearest organ**	**Side**	**Group**	**Organ detected/total number**	**Distance (mm)**	***p*-value**
From the needle shaft	Right	CQLB	7/7	4.7 ± 2.0	0.006
		NQLB	3/7	9.3 ± 0.9	
	Left	CQLB	5/7	4.5 ± 1.5	—
		NQLB	1/7	3.4 (single case)	
From the entry point	Right	CQLB	7/7	15.3 ± 6.0	0.001
		NQLB	7/7	27.6 ± 3.4	
	Left	CQLB	7/7	25.1 (3.3–30.5)	0.570
		NQLB	5/7	25.9 (4.7–32.9)	
From the needle tip	Right	CQLB	0/7	—	—
		NQLB	6/7	0.63 ± 0.23	
	Left	CQLB	2/7	0.53 ± 0.15	0.886
		NQLB	4/7	0.51 ± 0.17	

Data are presented as mean ± standard deviation or median (range), as appropriate. CQLB, conventional quadratus lumborum block; NQLB, novel quadratus lumborum block.

All 90° images in both groups on the right side contained at least one vital organ near the needle entry point. Here, the mean distance from the needle entry point to the nearest vital organ was significantly shorter in the CQLB group than in the NQLB group (*p* = 0.001). On the left side, seven and five images in the CQLB and NQLB groups, respectively, contained at least one vital organ near the needle entry point, with no significant between-group difference in the distance (*p* = 0.570).

On the right side, zero and six images in the CQLB and NQLB groups, respectively, contained at least one vital organ located distally to the needle tip. On the left side, two and four images in the CQLB and NQLB groups, respectively, contained at least one distal organ. No significant between-group difference was observed in the distance from the needle tip to the nearest organ on the left side (*p* = 0.886).

Table 3 summarizes the hypothetical needle entry points (on the tilted images) relative to the nearest vital organ. On the right side, no significant differences were found between groups at any angle. On the left side, there was a significant difference only at 100°, where the hypothetical entry point was closer to the vital organ in the CQLB group than in the NQLB group (*p* = 0.032).

**Table 3:** Number of images in which vital organs were detected and the distance from the hypothetical needle entry point, defined based on the actual entry point in the 90° image, on each tilted image.

**Side**	**Angle (°)**	**CQLB organ detected/total number**	**NQLB organ detected/total number**	CQLB distance to the nearest organ^*^(mm)	NQLB distance to the nearest organ^*^(mm)	***p*-value**
Right	70	7/7	6/7	15.0 ± 6.6	11.0 ± 8.3	0.379
	80	7/7	6/7	16.9 ± 9.8	14.8 ± 9.8	0.722
	100	7/7	6/7	7.5 ± 5.7	18.9 ± 13.1	0.086
	110	5/7	5/7	13.0 ± 10.5	18.4 ± 8.5	0.436
Left	70	7/7	5/7	16.3 ± 9.7	18.2 ± 11.7	0.765
	80	7/7	5/7	15.5 (5.0–29.8)	12.0 (3.5–27.4)	0.631
	100	6/7	5/7	16.1 (4.4–18.8)	26.2 (14.1–30.6)	0.032
	110	4/7	4/7	13.0 ± 10.5	18.4 ± 8.5	0.436

^*^Measured from the hypothetical entry point defined based on the 90° image.

Data are presented as mean ± standard deviation or median (range), as appropriate. CQLB, conventional quadratus lumborum block; NQLB, novel quadratus lumborum block.

Tables 4, 5 summarize the distribution of the nearest organs detected from the needle entry point, shaft, and tip on the 90° images, as well as from the hypothetical entry point on the tilted images. On the 90° images, blood vessels were the most frequently identified organs adjacent to the needle pathway in both groups. However, no organs were detected distal to the needle tip in any cat in the CQLB group, whereas blood vessels were identified distal to the needle tip in five of seven cats in the NQLB group on the right side (Table 4). On the tilted images, blood vessels were also the most commonly identified adjacent structures in both groups, while solid organs were infrequently observed and showed no consistent distribution pattern (Table 5).

**Table 4:** Distribution of the nearest organs to the needle entry point, shaft, and tip on the 90° ultrasonographic images.

**Side**	**Detection site of the nearest organ**	**Group**	**Blood vessel**	**Kidney**	**Spleen**	**Stomach**	**No organ**
Right	From the entry point	CQLB	7/7 (100%)	0/7 (0%)	0/7 (0%)	0/7 (0%)	0/7 (0%)
		NQLB	6/7 (86%)	1/7 (14%)	0/7 (0%)	0/7 (0%)	0/7 (0%)
	From the needle shaft	CQLB	6/7 (86%)	1/7 (14%)	0/7 (0%)	0/7 (0%)	0/7 (0%)
		NQLB	7/7 (100%)	0/7 (0%)	0/7 (0%)	0/7 (0%)	0/7 (0%)
	From the needle tip	CQLB	0/7 (0%)	0/7 (0%)	0/7 (0%)	0/7 (0%)	7/7 (100%)
		NQLB	5/7 (71%)	0/7 (0%)	0/7 (0%)	0/7 (0%)	2/7 (29%)
Left	From the entry point	CQLB	6/7 (86%)	0/7 (0%)	1/7 (14%)	0/7 (0%)	0/7 (0%)
		NQLB	5/7 (71%)	1/7 (14%)	1/7 (14%)	0/7 (0%)	0/7 (0%)
	From the needle shaft	CQLB	3/7 (43%)	0/7 (0%)	2/7 (29%)	0/7 (0%)	2/7 (29%)
		NQLB	2/7 (29%)	0/7 (0%)	1/7 (14%)	0/7 (0%)	4/7 (57%)
	From the needle tip	CQLB	4/7 (57%)	0/7 (0%)	0/7 (0%)	0/7 (0%)	3/7 (43%)
		NQLB	5/7 (71%)	0/7 (0%)	0/7 (0%)	0/7 (0%)	2/7 (29%)

CQLB, conventional quadratus lumborum block; NQLB, novel quadratus lumborum block.

**Table 5:** Distribution of the nearest organs to the hypothetical needle entry point, defined according to the actual entry point on the 90° image, for each tilted image.

**Side**	**Angle (°)**	**Group**	**Blood vessel**	**Kidney**	**Spleen**	**Stomach**	**No organ**
Right	70	CQLB	5/7 (71%)	2/7 (29%)	0/7 (0%)	0/7 (0%)	0/7 (0%)
		NQLB	5/7 (71%)	2/7 (29%)	0/7 (0%)	0/7 (0%)	0/7 (0%)
	80	CQLB	6/7 (86%)	1/7 (14%)	0/7 (0%)	0/7 (0%)	0/7 (0%)
		NQLB	6/7 (86%)	1/7 (14%)	0/7 (0%)	0/7 (0%)	0/7 (0%)
	100	CQLB	5/7 (71%)	1/7 (14%)	1/7 (14%)	0/7 (0%)	0/7 (0%)
		NQLB	6/7 (86%)	1/7 (14%)	0/7 (0%)	0/7 (0%)	0/7 (0%)
	110	CQLB	5/7 (71%)	1/7 (14%)	0/7 (0%)	0/7 (0%)	1/7 (14%)
		NQLB	5/7 (71%)	1/7 (14%)	0/7 (0%)	0/7 (0%)	1/7 (14%)
Left	70	CQLB	5/7 (71%)	2/7 (29%)	0/7 (0%)	0/7 (0%)	0/7 (0%)
		NQLB	4/7 (57%)	2/7 (29%)	1/7 (14%)	0/7 (0%)	0/7 (0%)
	80	CQLB	5/7 (71%)	2/7 (29%)	0/7 (0%)	0/7 (0%)	0/7 (0%)
		NQLB	4/7 (57%)	2/7 (29%)	1/7 (14%)	0/7 (0%)	0/7 (0%)
	100	CQLB	4/7 (57%)	2/7 (29%)	1/7 (14%)	0/7 (0%)	0/7 (0%)
		NQLB	3/7 (43%)	2/7 (29%)	2/7 (29%)	0/7 (0%)	0/7 (0%)
	110	CQLB	3/7 (43%)	0/7 (0%)	2/7 (29%)	1/7 (14%)	1/7 (14%)
		NQLB	2/7 (29%)	0/7 (0%)	2/7 (29%)	1/7 (14%)	2/7 (29%)

CQLB, conventional quadratus lumborum block; NQLB, novel quadratus lumborum block.

## 4 Discussion

The proximity of the needle trajectory to abdominal or retroperitoneal organs in CQLB increases the risk of organ injury (11, 12). Accordingly, we investigated the relationship between the needle trajectory (namely, the needle shaft) and adjacent organs in both the NQLB and CQLB approaches. Furthermore, inadequate visualization of the needle tract or an incorrect needle angle during ultrasound-guided interventions increase the risk of inadvertent injury to surrounding structures (14–16). Therefore, we also aimed to investigate the distance from the needle entry point and tip to adjacent organs on the 90° image as well as the presence of vital organs in tilted images to assess the potential risk of organ injury caused by misdirected needle advancement.

The safety profile of the NQLB is reflected by the generally low rate of needle trajectories located proximal to vital organs, which was indicated by a distance of < 5 mm from the needle shaft. Given that there remains no established recommendation regarding a safe distance between the needle trajectory and adjacent organs in regional anesthesia, we arbitrarily selected the 5-mm threshold for analytical purposes. However, a recent study on needle trajectory optimization for percutaneous biopsy procedures demonstrated the feasibility and safety of maintaining a minimum distance of approximately 3.1–3.3 mm from critical structures (17). Accordingly, we adopted a slightly larger margin to ensure an additional safety buffer. Although a lower proportion of organs located within 5 mm of the needle trajectory is not an absolute safety indicator, it is reasonable to consider that the NQLB yielded a relative safety margin. In the CQLB group, a relatively small number of needles passed close to vital organs on the left side. This finding suggests that the potential risk of organ injury in CQLB may differ between the right and left sides. A previous anatomical study has demonstrated cranio-caudal asymmetry in renal positioning in cats, with the left kidney more frequently located at the L3–L5 vertebral levels (18). This anatomical characteristic may have influenced the spatial relationship between the predefined needle trajectory and adjacent organs on the left side in the present study. This suggests that the potential risk of organ injury in CQLB may depend on anatomical differences between the right and left sides. However, QLB is commonly performed bilaterally given that it is primarily used for celiotomy procedures due to its ability to block visceral afferent nerves (3–10). Taken together, our findings indicate a safety advantage of NQLB with respect to the risk of inadvertent organ injury, particularly on the right side, where a significant difference was observed. By contrast, on the left side, no definitive conclusion can be drawn regarding relative safety between the two approaches in this study.

Furthermore, the NQLB yielded a safe distance between the needle entry point and the nearest vital organ. This suggests that this novel technique allows both safe and adjustable needle insertion, in addition to the aforementioned safe needle pathway.

However, our findings suggest that NQLB may be associated with a greater proximity to distal organs, as vital structures, particularly blood vessels, were more frequently observed distal to the needle tip. This observation may be related to the anatomical characteristics of the QLB target region, including the quadratus lumborum and psoas muscles, which are located deep within the body and in close proximity to major vascular structures. Consequently, even in the novel approach, the spatial distance between the advancing needle tip and adjacent blood vessels may decrease. Geometrically, the two approaches differ in their needle pathways relative to adjacent vascular structures. In the NQLB, the needle trajectory is directed toward regions where blood vessels are more likely to be encountered near the target site, whereas in the CQLB, vascular structures are more commonly encountered along the needle shaft during advancement toward the target. This geometric difference may have influenced whether vascular structures were identified distal to the needle tip or along the needle shaft. Contrary to our hypothesis, we observed no significant between-group difference in the distance from the hypothetical needle entry point, which was extrapolated from the actual entry point, to the nearest organ on the tilted images, except for the 100° image on the left side. These findings suggest that deviations from the intended insertion trajectory, such as excessive needle advancement or an incorrect insertion angle, may influence the spatial relationship between the needle and adjacent organs under certain conditions. Therefore, a precise angle of insertion and accurate needle placement at the target site are essential even during the NQLB.

Inconsistent with previous cadaveric studies (11, 12), blood vessels such as the aorta and caudal vena cava were the most frequently identified adjacent to the needle pathway in both groups. In our study, the ultrasound probe was compressed against the body trunk to optimize the needle insertion pathway. This maneuver may have displaced mobile organs such as the kidney and spleen, resulting in fewer instances of these organs being located near the needle shaft or entry point. Accordingly, the aforementioned discrepancy can be attributed to probe compression and differences in body composition between living animals and frozen cadaveric specimens, with freeze–thaw processing altering the mechanical behavior of abdominal organs (19, 20). Nevertheless, major blood vessels, namely the aorta and caudal vena cava, tend to remain close to the needle in both techniques. Therefore, particular attention should be directed toward these structures during both QLB procedures.

This study has some limitations. First, the sample size was relatively small. The sample size was determined based on the results of a pilot study, which demonstrated an apparent difference in the proportion of cases with organs located close to the needle trajectory, similar to our findings. Nevertheless, the limited sample size may have contributed to potential type II errors, particularly in the secondary outcomes. Second, assessors were not blinded given the apparent between-technique differences. Furthermore, all measurements were performed by the same researcher who conducted the ultrasound-guided needle insertions given that there were no additional veterinarians available other than those involved in performing the QLB procedures and storing the ultrasonographic images. Although this limitation may have introduced observer bias, the objective and distance-based nature of the measurements was considered to minimize its impact on the results. Third, the potential risk of needle-related organ injury should ideally be evaluated using three-dimensional imaging given that two-dimensional ultrasonography provides limited information regarding organs located parallel to the needle (21, 22). However, since we included clinical cases, we did not perform additional imaging procedures such as computed tomography due to ethical considerations given the resulting unnecessary radiation exposure or prolonged anesthetic duration. To partially overcome this limitation, tilted images were captured and analyzed to estimate the potential risk of organ contact along the plane parallel to the needle. Therefore, further studies are warranted to investigate the three-dimensional spatial relationship between the needle and adjacent organs. Fourth, our findings cannot readily be generalized to lumbar vertebral levels other than those targeted herein. We enrolled cats scheduled for elective ovariohysterectomy to ensure a relatively homogeneous population undergoing a standardized surgical procedure. Moreover, the transverse process of the second lumbar vertebra in the CQLB and the intertransverse space between the second and third lumbar vertebrae in the NQLB were selected as the target sites in accordance with previous similar studies (4–7, 13). Further studies targeting other lumbar levels are warranted to extend the applicability of the NQLB technique.

## 5 Conclusion

The NQLB provided safer positioning of the needle trajectory and needle entry point by increasing the distance from nearby vital organs compared with the CQLB; this outcome indicates its potential in lowering the risk of inadvertent organ injury during QLB in cats. By contrast, with respect to needle tip placement, the CQLB may offer a relative safety advantage once the target site is reached, owing to a greater separation between the needle tip and adjacent organs. However, real-time ultrasound visualization of the entire needle path remains essential to avoid inadvertent injury to structures located distal to the target site and to minimize the risks associated with misdirected needle advancement. Future studies are necessary to elucidate the three-dimensional anatomical relationship between the needle and adjacent organs, investigate its characteristics at other lumbar levels, and identify potential complications in clinical cases.

## Data Availability

The raw data supporting the conclusions of this article will be made available by the authors, without undue reservation.
